# Testing Pancreatic Islet Function at the Single Cell Level by Calcium Influx with Associated Marker Expression

**DOI:** 10.1371/journal.pone.0122044

**Published:** 2015-04-08

**Authors:** Jennifer H. R. Kenty, Douglas A. Melton

**Affiliations:** 1 Department of Stem Cell and Regenerative Biology, Harvard University, Harvard Stem Cell Institute, Cambridge, Massachusetts, United States of America; Virgen Macarena University Hospital, School of Medicine, University of Seville, SPAIN

## Abstract

Studying the response of islet cells to glucose stimulation is important for understanding cell function in healthy and disease states. Most functional assays are performed on whole islets or cell populations, resulting in averaged observations and loss of information at the single cell level. We demonstrate methods to examine calcium fluxing in individual cells of intact islets in response to multiple glucose challenges. Wild-type mouse islets predominantly contained cells that responded to three (out of three) sequential high glucose challenges, whereas cells of diabetic islets (db/db or NOD) responded less frequently or not at all. Imaged islets were also immunostained for endocrine markers to associate the calcium flux profile of individual cells with gene expression. Wild-type mouse islet cells that robustly fluxed calcium expressed β cell markers (INS/NKX6.1), whereas islet cells that inversely fluxed at low glucose expressed α cell markers (GCG). Diabetic mouse islets showed a higher proportion of dysfunctional β cells that responded poorly to glucose challenges. Most of the failed calcium influx responses in β cells were observed in the second and third high glucose challenges, emphasizing the importance of multiple sequential glucose challenges for assessing the full function of islet cells. Human islet cells were also assessed and showed functional α and β cells. This approach to analyze islet responses to multiple glucose challenges in correlation with gene expression assays expands the understanding of β cell function and the diseased state.

## Introduction

Techniques to assess pancreatic β cell function are required for developing β cell replacement therapies for diabetics. Cells generated *ex vivo*, *e*.*g*. human β cells derived from the directed differentiation of pluripotent stem cells, need to be validated by various methods to assess physiological function. Antibody staining, histology, transcriptional and proteomic assays, and glucose stimulated insulin secretion (GSIS) assays are widely used to assess β cells or islets [1–5]. Yet a major limitation of these techniques is the lack of temporal data, particularly with regards to the activity of single cells. Assays that test the live, real-time function of individual islet cells or β cells will deepen understanding of the cellular dysfunction that occurs in diabetes.

Previous studies have demonstrated the utility of calcium imaging in whole pancreatic islets. Calcium influx in islets has been correlated with oxygen consumption rate [6], nuclear calcium oscillation [1], metabolic oscillation of NAD(P)H [7], and glucose stimulated insulin secretion profiles [8]. And the sodium-calcium exchange protein NCX1 and its relationship to improved insulin secretion and glucose sensitivity has been effectively studied using patch clamp and calcium imaging in islets [9]. Defective calcium influx in diabetic islets was demonstrated in the islets of db/db mice by analyzing the calcium flux level in whole, intact islets [10].

Assaying the function of islets at a single cell level with temporal resolution has recently become possible with electrophysiological measurements and calcium imaging techniques that have fuelled advancements in neural and endocrine physiology [11–19]. Studies have demonstrated calcium imaging in a few pancreatic islet cells and provided crucial information on the calcium influx pattern in different islet cell types [20–23]. More recently, calcium flux of individual cells in sectioned WT mouse islets was analyzed using laser scanning confocal microscopy and this showed that beta cells are connected in highly efficient connectivity networks [24, 25]. This study, done on sectioned islets, shows the importance of testing function in whole (undispersed) islets. Hodson et al. have extended this functional connectivity analysis on intact islets and analyzed the response to incretins in response to diabetogenic lipotoxicity [26].

Here we extend these approaches and demonstrate a way to analyze the function of single cells in whole islets in response to multiple sequential glucose challenges. The cellular function is analyzed with temporal and spatial resolution and is correlated with gene expression to identify the cell types. This approach focuses on the robustness of a cell’s physiological response and provides a quantitative measurement of individual islet cell function.

## Materials and Methods

### Ethics Statement

Animal studies were performed in strict accordance with the recommendations in the Guide for the Care and Use of Laboratory Animals of the National Institutes of Health. All of the animals were handled according to approved institutional animal care and use committee (IACUC) protocol number [13–15]. The protocol was approved by the Committee on the Use of Animals in Research and Teaching of Harvard University Faculty of Arts & Sciences (HU/FAS). The HU/FAS animal care and use program is AAALAC International accredited, has a PHS Assurance (A3593-01) on file with NIH’s Office of Laboratory Animal Welfare, and is registered with the USDA (14-R-0128). Animals were euthanized in accordance with AVMA Guidelines for the Euthanasia of Animals. Rodent Islet isolation protocol was used in this study to procure viable and functional islets from mouse pancreas. Human pancreatic islets from non-diabetic donors were obtained through Prodo Laboratories with appropriate consent. All tissue samples were rendered anonymous. Work with the human material used in this project was reviewed by the Committee on the Use of Human Subjects (the Harvard IRB) and was determined to be not human subjects research.

### Mice and islet sources

Mouse strains, ICR, db/db and NOD mice were obtained from Jackson Laboratories. The ages of the diabetic mice used are as follows: NOD mice (NOD/SchiLtJ from Jackson Laboratory) were 12–13 week old female mice. db/db mice (B6.BKS(D)-^*Leprdb*^/J from Jackson Laboratory) were 11–12 week old male mice. Mouse islets were freshly isolated as previously described [27] after verifying the fed blood glucose level was above 550 mg/dL for db/db and NOD mice.

### Islet plating for imaging and glucose challenges

After isolation, islets were picked for imaging based on minimal background autofluorescence signals, since high background fluorescence may occur due to exocrine contamination [24]. Islets were plated at 10–20 islets/well into the wells of a 96-well plate (Sigma; CLS3904) that had been coated with 804G-conditioned media and incubated overnight at 37°C, 5% CO_2_ in islet media (RPMI1640 (Sigma; R0883), 10% (vol/vol) FBS serum (Valley Biomedical; BS3033), 1× penicillin/streptomycin (Invitrogen; 15070–063), 1× Glutamax (Invitrogen; 35050–079)). During islet plating, islets were concentrated to the center of the well by flushing with buffer using a 200uL pipette to position the islets. This step is important for maximizing the number of islets in the field of view of the microscope. For Fluo-4 AM dye (Life Technologies; F-14201) loading, serum-containing media was removed and replaced with fasting buffer (2.5mM glucose in KRB solution:128 mM NaCl, 5 mM KCl, 2.7 mM CaCl_2_, 1.2 mM MgSO_4_, 1 mM Na_2_HPO_4_, 1.2 mM KH_2_PO_4_, 5 mM NaHCO_3_, 10 mM HEPES (Life Technologies; 15630080), 0.1% BSA (Proliant; 68700) in deionized water). Prior to imaging, islets were incubated with Fluo-4 AM in fasting buffer at 37°C, 5% CO_2_ for 45 minutes followed by four washes in dye-free fasting buffer over 15 minutes. Media changes were done by hand-aspirating the media using a pipetteman and gently adding warmed buffer (37°C) back into the well by pipetting on the side to minimize stress to the islets and prevent detachment of the islets.

For sequential glucose stimulations during calcium imaging, islets were first incubated in low glucose fasting buffer (2.5 mM glucose in KRB) for 5 minutes, then changed to high glucose buffer (15 mM glucose in KRB) for 5 minutes, followed by serial low-high-low-high glucose with a total of 3 high glucose challenges over 30 minutes. The final step was addition of 30 mM KCl in low glucose buffer for 5 minutes to depolarize the cells. All glucose and KCl solutions added to islets were warmed to 37°C before adding to the islets.

### Imaging Microscopy

An AxioZoom V16 microscope (Carl Zeiss) was used to acquire high resolution time series images. This microscope provides a large field of view that enables the simultaneous imaging of a large region of the specimen such that the whole surface of a single islet can be in focus with a 70X objective. Imaging was done with a 30X objective for a population analysis of the multiple islets and a 70X objective for single cell level analyses of a single islet. Eighteen images were taken during each 5 minutes glucose challenge (every 17 seconds) and a total of 126 images were acquired during each series of three glucose challenges. Before imaging, islets were switched to fresh low glucose solution to remove any possible insulin contamination from the fasting step. The maximum number of wells imaged during one imaging run was 8 wells. Time spent to change solution in one well was about 10 seconds, with a maximum time spent changing solution of 80 seconds. The imaging target was focused by setting the x, y and z planes for each islet to be imaged. The first images of each imaging target were taken by automatic snap shot that occurred every second. The second images were taken 17 seconds after the first snap shot. Imaging was stopped after 18 images were taken for 5 minutes. Then low glucose solution was changed to high glucose solution for up to 8 total wells for 80 seconds maximum. The focus setting of x, y, z planes were confirmed briefly within 20 seconds and the same 5 minute imaging was repeated. The total volume of the solution in the wells stayed constant (100uL per well) during glucose solution change in order to prevent light diffraction during imaging which may blur the images. Buffer changes for glucose challenges were done by hand-aspirating, as described above. Between imaging, glucose solution changes were done without the wash step to reduce the time lag for imaging and to minimize stress to the islets.

Acquired images were stacked, converted to a movie in avi format, and played at 10 frames per second using ImageJ/Fiji imaging analysis program. Movies are available as supporting information.

### Immuno-Fluorescent Staining

Post calcium imaging, calcium imaged islets were fixed by immersion in 4% PFA for 20 minutes at room temperature (RT). Samples were washed 3 times with PBS + 0.3% Triton X-100 (VWR; EM-9400) (PBST) at RT, followed by incubation in primary antibodies overnight at 4°C. The following primary antibodies diluted in PBS were used: guinea pig anti-insulin (Dako; A0564) (1:100), mouse anti-Nkx6.1 (University of Iowa, Developmental Hybridoma Bank; F55A12-supernatant) (1:200), and rabbit anti-glucagon (Abcam; ab92517) (1:200). After primary antibody incubation, islets were washed 3 times in 0.3% PBST, followed by secondary antibody incubation for 1 hour at RT. Secondary antibodies conjugated to Alexa Fluor 488, 594, and 647 were diluted (1:300) in PBS and used to visualize primary antibodies. Stained islets were then imaged again using AxioZoom V16 Microscope at a 70X objective with the same image plane (x, y, and z) used in prior calcium imaging. Three images total, each showing the staining of INS, GCG, or NKX6.1, were added to a stack with the calcium time series images, and islets in the images were aligned by StackReg application for the imaging analysis.

### Image Analysis

Analysis of the time series recorded calcium flux and immuno-fluorescence stain image stack was performed using ImageJ /Fiji. StackReg application in ImageJ/Fiji was used to correct for the subtle movement of the islets over the course of the imaging. The ROI manager in ImageJ/Fiji was used to manually draw circles around the islets or individual cells to measure the average fluorescent intensity (quantified as average area under the curve, or ave a.u.c.) within the selected area. Individual cells were selected for single cell analysis if they showed at least minimal calcium staining in low glucose buffer. A two-tailed distribution and paired t-test was used to compare the average area under the curve (a.u.c.) during low glucose and high glucose stimulation, and the calcium influx response was defined as positive when the P-value was < 0.05, with the ave a.u.c. of high glucose higher than the ave a.u.c of low glucose. Positive calcium responses for second and third glucose challenges were noted only when preceding ave a.u.c of high glucose was higher than the subsequent ave a.u.c of low glucose. Immunostaining signal was defined positive when the average fluorescence intensity of ROI manager selection was higher than average fluorescence intensity of background signals plus two standard deviations.

### Methodological considerations

Accounting for subtle morphology changes of the islets during imaging and accurate aligning of calcium images for temporal resolution are important factors to consider for reliable single cell analysis. Past studies have accounted for this issue by rejecting the trace of the single cell from the time series images if extensive motion artifacts were observed [24, 25]. The calcium dye signal to background is not large enough to utilize software-dependent automatic selection of cells based on this intensity ratio (an option in ImageJ/Fiji software). Alternatively, manual tracking and measurement of fluorescent intensity changes and marker expression of every cell within the islet over 126 calcium images and 3 immunostained images was accomplished by manually select cells from one representative image and confirm by eye that the selection applies to all 129 images. The stacking of 129 images, selecting single cells on the first stack image, and confirming the selections with the rest of 128 stack images were done to make the tracking process efficient with the added benefit that selections can be confirmed while observing the time series movie. Manual verification of alignment of islets in the stack before and after the selection is important for obtaining reliable and accurate analysis results. In our experience, unstacking the 7 time series calcium imaging and immunostain images prior to stacking all the images (which is automatically done by the Axiozoom 16V software Zen) provided good alignment of the images.

## Results

### A Method to Measure Calcium Flux in Islets in Response to Sequential Glucose Challenges

**Fig 1:**
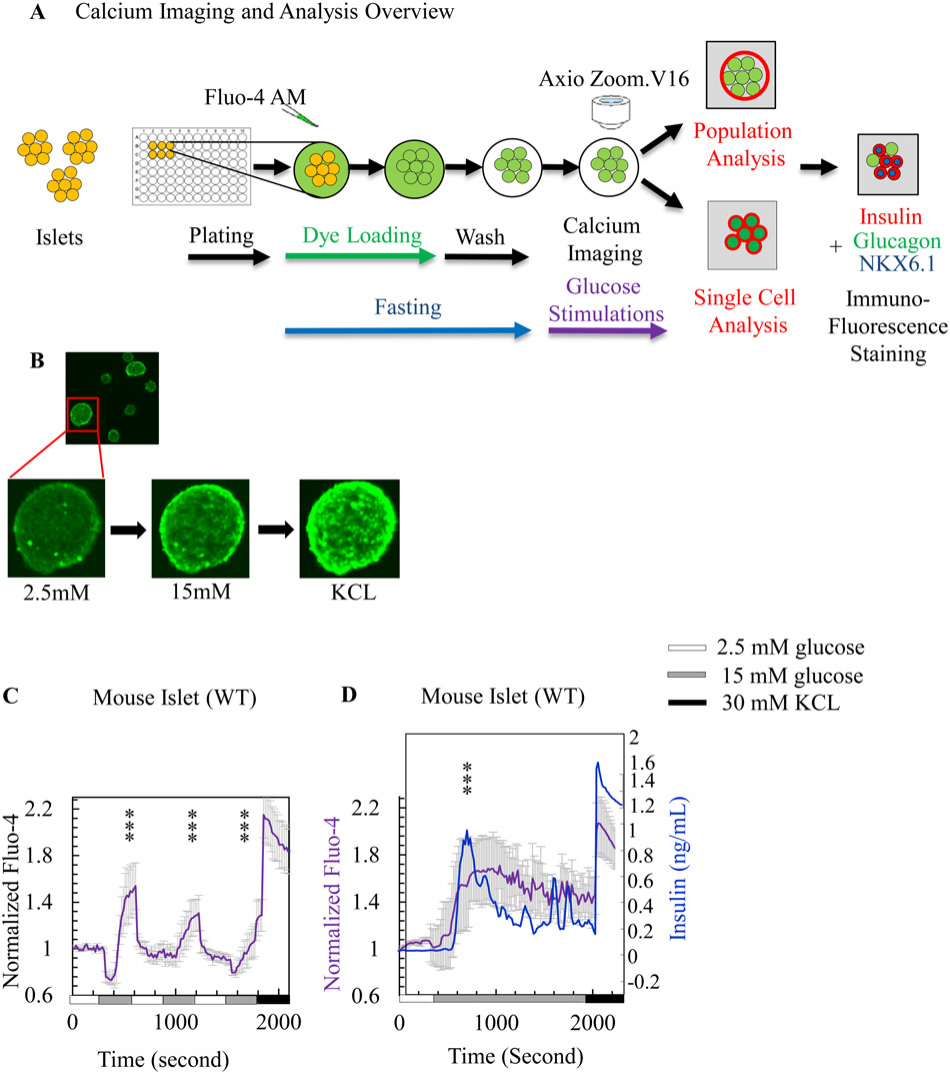
A rise in intracellular calcium corresponds to insulin secretion. (A) Overview of calcium imaging method for islets: intact islets were plated on a 96 well plate. Islets were stained with the Fluo-4 AM and then washed in fasting solution. Calcium influx of the population of cells in the islet or single cells was imaged following the addition of glucose. Same islets were imaged again after fixation and immunofluorescence staining with INS, GCG, and NKX6.1. (B) Representative images of WT mouse islets after stimulating with 2.5 and 15 mM glucose followed by 30 mM KCl. (C) Average normalized population measurements with standard deviation of dynamic Fluo-4 fluorescence intensity for WT mouse islets shown in Fig 1B (7 islets) with corresponding S1 Movie. The calcium influx response for each mouse islet was normalized to the starting fluorescence intensity data point during the initial low glucose incubation. The normalized fluorescence intensities of 7 WT mouse islets were averaged and plotted on the y-axis with standard deviations. Islets were challenged sequentially with 2.5, 15, 2.5, 15, 2.5, and 15 mM glucose and 30 mM KCl. Fluorescence was measured at 126 time points throughout the series of glucose challenges, normalized to the starting fluorescence intensity, and averaged across all islets at each time point. The standard deviation at each time point ranged between ± 2 to 8 a.u.c (area under the curve). The x-axis represents time (in seconds). P-value was calculated from the difference between ave a.u.c. during low glucose and high glucose stimulations. Significance of calcium influx response was labeled * when the P-value was between 0.05 and 0.001 and ** when the P-value was below 0.001. (D) Average normalized population measurements with standard deviation of dynamic Fluo-4 fluorescence intensity of a total of eight WT mouse islets with corresponding S2 Movie. Population measurements of dynamic normalized Fluo-4 fluorescence intensity for mouse islets is shown in purple, and the ELISA measurements of secreted mouse insulin for the same batch of islets is shown in blue. Challenges were done with 5 minutes of 2.5 mM, 25 minutes of 15 mM, and 5 minutes of 30 mM KCl.

Towards the goal of assessing the function of healthy and diseased islets, a method was developed to image calcium flux of whole islets (population analysis) and individual islet cells (single cell analysis) in intact islets followed by fixation and immunostaining to examine marker expression profiles of calcium-imaged cells (Fig 1A). Purified islets were loaded with the indicator, Fluo-4 AM, which fluoresces upon binding calcium. Islets were imaged while being stimulated with a series of sequential glucose challenges: low (2.5 mM)-high (15 mM)-low-high-low-high followed by KCl depolarization. Images were collected during glucose challenge to generate image stacks that were used to quantify the calcium signal at the population and single cell level over time. Representative images of the peak calcium response to low glucose, high glucose, and KCl for a mouse islet are shown in Fig 1B. Fluo-4 dye penetrates the first and second cellular layers of intact islets [26]. The fluorescent signal from cells at the edge of the islets (15 mM glucose and KCl treatments, Fig 1B) appears brighter because cells in the middle, located in the z-plane, are not in focus.

A population analysis of WT mouse islets shows calcium influx in response to high glucose stimulations and KCl (Fig 1C and corresponding S1 Movie). The fluorescence intensity rises dramatically approximately five minutes following the addition of the first high glucose challenge. The fluorescence intensity initially dips in the baseline signal that immediately coincides with changing to the high glucose buffer, which could be caused by changes in intracellular calcium levels or the buffer change reducing the baseline level of calcium dye. Calcium fluxing was also observed after the second and third high glucose challenges, but with lower peaks compared to the first challenge. It is unknown if the declining calcium influx responses are a natural islet response to multiple glucose challenges or are only observed *in vitro*.

An extended high glucose challenge was performed to compare the kinetics of calcium flux to insulin secretion from the same WT mouse islet batch (Fig 1D and S2 Movie). Population analysis shows a calcium spike that corresponded to insulin secretion with calcium influx preceding insulin secretion as reported previously [8]. First phase insulin secretion peaks about two minutes after the calcium peak, or about 7 minutes after initiating high glucose. Second phase insulin secretion occurred 13–25 minutes after the addition of high glucose. During this second phase response, steady-state oscillations or bursting calcium responses were observed [2]. The near-overlapping, biphasic responses of calcium influx and insulin release indicate that calcium burst correlates to insulin secretion. This result is consistent with oscillations in calcium influx acting as the principal driver of islet insulin secretion [10, 28–31].

### Population and Single Cell Based Calcium Influx Analysis Reveal Defects in Diabetic Mouse Islets

**Fig 2:**
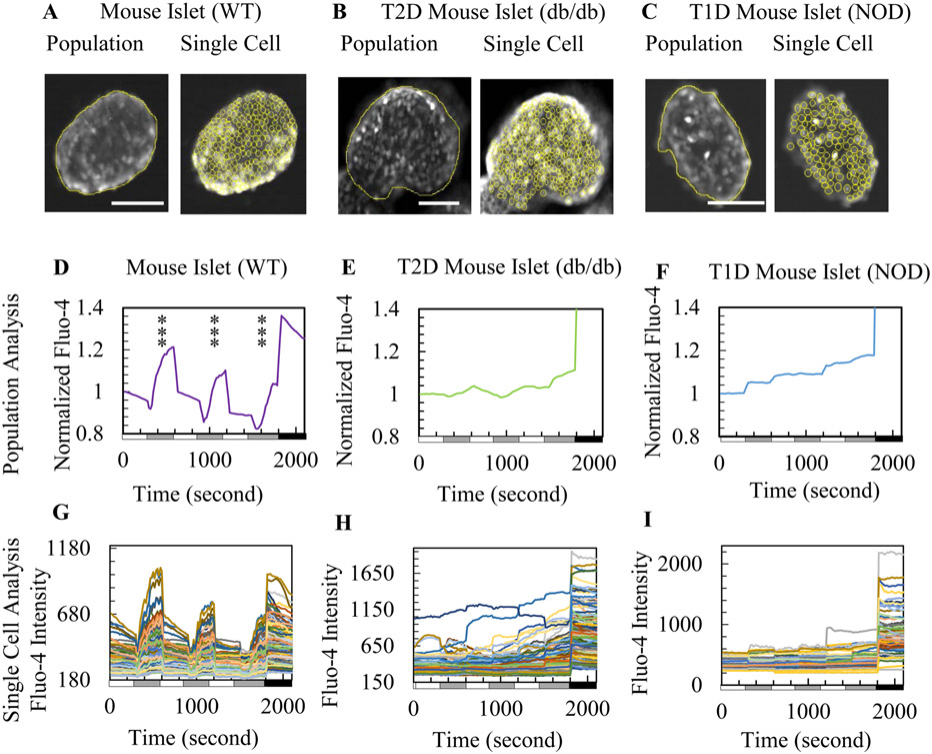
Population and single cell-based calcium influx analysis show defects in diabetic mouse islets. (A-C) Representative images of analysis selection setting for population (left) and single cell (right) analysis for (A) WT mouse islet, (B) db/db mouse islet, and (C) NOD mouse islet. Scale bar = 100 μm. Note: Fed blood glucose level of the db/db and NOD mice was > 550 mg/dL. (D-F) Population measurements of dynamic normalized Fluo-4 fluorescence intensity for one islet (out of three islets analyzed for each mouse strain): (D) WT mouse islet, (E) db/db mouse islet, and (F) NOD mouse islet calcium imaging during sequential glucose stimulation. (G-I) Single cell measurements of dynamic Fluo-4 fluorescence intensity for (G) WT mouse islets, (H) db/db mouse islets, and (I) NOD mouse islets upon calcium imaging during glucose challenges.

Calcium imaging was utilized to compare glucose-stimulated calcium influx in the islets of WT and diabetic mice at the population and single cell levels (Fig 2). WT islets were obtained from normal ICR mice, Type II diabetic islets were obtained from db/db mice, and Type I diabetic islets were obtained from NOD mice. Fig 2A–2C show the population and single cell analysis of calcium imaging. WT islets fluxed calcium in response to high glucose at the population level (Fig 2D) and with well-synchronized responses among single cells (Fig 2G and S3 Movie). In contrast, db/db and NOD islets showed weak calcium responses to glucose stimulation at the population level (Fig 2E, 2F and S4 Movie) and lack the synchronous phenotype observed at the single cell level (Fig 2H, 2I and S5 Movie). A comparison of Fig 2D–2G to Fig 2E–2I shows a clear defect in the diabetic islet cells with respect to dynamic and coordinated calcium flux.

**Fig 3:**
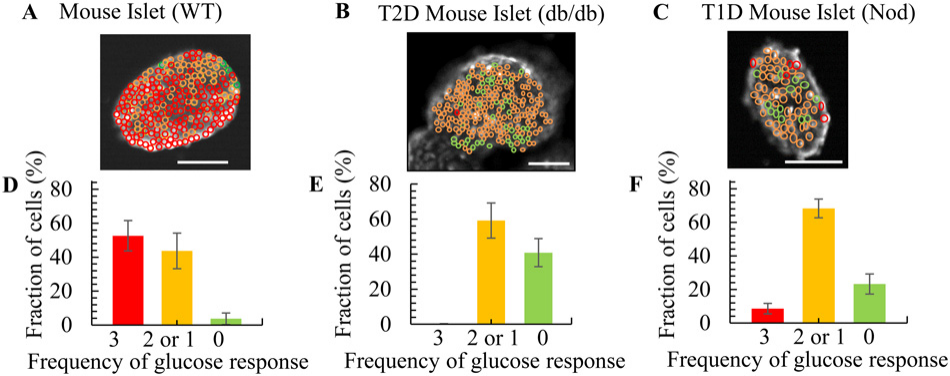
Single cell based calcium influx analysis reveals a quantitative difference in glucose responsive cells between WT and diabetic mouse islets. (A-C) Representative images showing the number of single cells that responded to 3 (red), 2 or 1 (orange), and 0 (green) glucose challenges in (A) WT mouse islets, (B) db/db mouse islets, and (C) NOD mouse islets. (D-F) Quantification of the frequency of cells responding to 15 mM glucose analyzed from 3 islets: (D) WT mouse islet cells (total number of cells analyzed from each islet was n = 216, n = 190, n = 144), (E) db/db mouse islet cells (n = 239, n = 132, n = 113), and (F) NOD mouse islet cells (n = 69, n = 64, n = 50). The WT islets had on average 53±9% of fully responsive cells and 4±3% of non-responsive cells, while db/db and NOD islets on average had 1±1% and 9±3% fully responsive cells and 59±10% and 23±6% non-responsive cells accordingly. Scale bar = 100 μm.

Calcium responsiveness of individual cells was assessed based on the number of responses to three glucose challenges (Fig 3). Cells that flux calcium in response to all three glucose stimulations are circled in red, cells that responded once or twice are circled orange, and cells that did not respond are circled green (Fig 3A–3C). For WT mouse islets, there was a large proportion of more responsive red and orange cells positioned close to each other and the few non-responsive green cells were typically found at the islet’s edge (Fig 3A). In comparison, db/db and NOD islets had random positioning of red, orange, and green cells (Fig 3B and 3C). WT islets predominantly contained cells that responded to all three glucose stimulations (Fig 3D), whereas cells from diabetic islets responded to glucose less frequently or not at all (Fig 3E and 3F).

### Marker expression analysis demonstrates diabetic islets contain β and α cells with aberrant calcium responses

**Fig 4:**
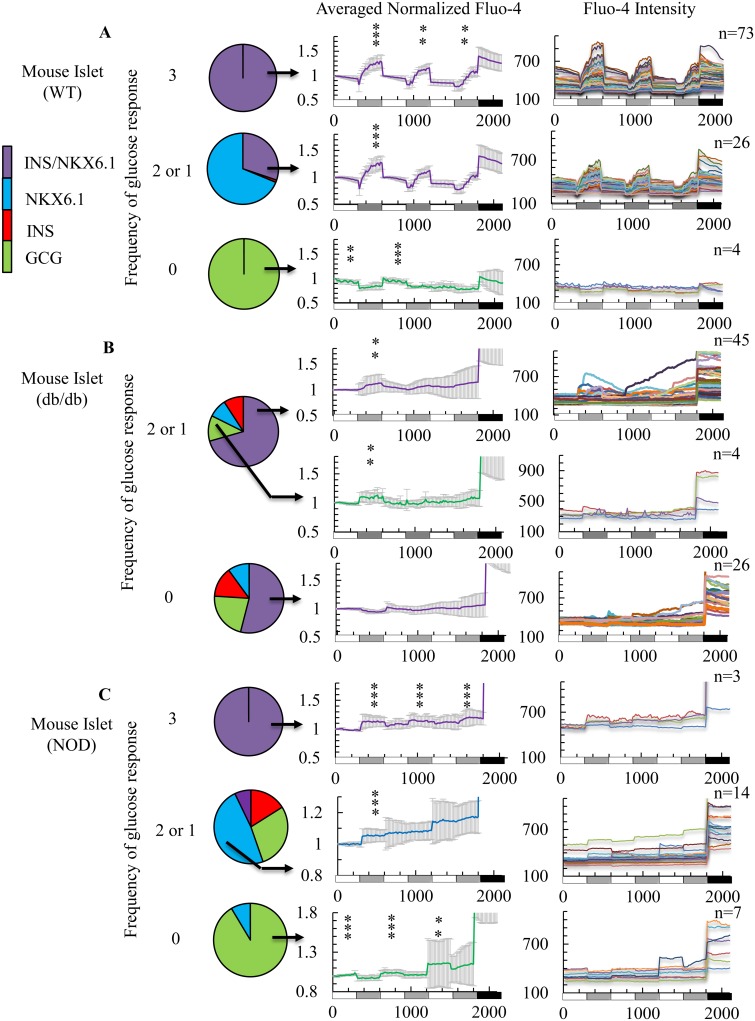
Analysis of islet cell calcium response with staining for endocrine markers allows further characterization of WT and diabetic islets. Color scheme for the list of markers analyzed is on the left panel; INS/NKX6.1 (Purple), NKX6.1 (Blue), INS (Red), and GCG (Green). Pie charts on the right panel show the composition of marker expression with the indicated frequency of glucose responsiveness for (A) WT, (B) db/db, and (C) NOD mouse islets. Average normalized single cell measurements with standard deviation of dynamic Fluo-4 fluorescence intensity on the left graphs. Single cell measurements of dynamic Fluo-4 fluorescence intensity on the right graphs.

Imaged islets were fixed and stained for endocrine cell markers to correlate calcium influx response to cell identity (see the images in S1 Fig, pie chart in Fig 4, and complete raw data in S1 Table). Calcium-imaged cells were assessed for four marker gene profiles (INS/NKX6.1, NKX6.1, INS, and GCG) and compared marker expression to glucose responsiveness (3, 1 or 2, or 0 times) at the single cell level. WT mouse islet cells that responded to all 3 high glucose stimulations were almost entirely cells expressing markers of mature β cells (INS/NKX6.1, Fig 4A, top panel). Cells of WT islets that responded to only 2 or 1 glucose challenges expressed INS/NKX6.1 or NKX6.1 only (Fig 4A, middle panel). Cells that did not respond to glucose showed an inversed calcium trace and expressed the α cell marker, GCG (Fig 4A, bottom panel). These data show that the most glucose-responsive cells coexpress INS/NKX6.1, but about 30% of co-positive INS/NKX6.1 cells, do not respond to every glucose challenge (Fig 4A and S1 Table). Calcium imaging and immunostaining of a representative cell within WT islets that showed a strong response to all high glucose challenges (β cell) and a cell that showed an inverse response (α cell) are presented in S2 Fig, and movies of the same healthy α cell and β cell are shown in Movies S6 Movie and S7 Movie.

Notably, diabetic mouse islet cells did not contain functional β cells, defined as cells that responded multiple times to glucose stimulation as observed for WT islets. The db/db islets did not contain any cells that responded to all three glucose challenges but did have partially-responsive (2 or 1) or non-responsive cells that expressed markers of β cells (INS/NKX6.1) (Fig 4B and S1 Table). The NOD islets contained small numbers of cells that responded to all three glucose challenges and expressed β cell markers (INS/NKX6.1), but the calcium responses of these cells were not as robust or synchronized as functional β cells seen in WT mouse islets. For NOD islets, the majority of partially-responsive cells expressed NKX6.1, and non-responsive cells expressed GCG (Fig 4C and S1 Table). Interestingly, most failed calcium influx responses to high glucose were observed during the second and third glucose challenges. Both db/db and NOD mouse islet cells contained dysfunctional α cells (based on GCG expression) that inappropriately responded to high glucose challenges (Fig 4B and 4C). Two representative dysfunctional α and β cells that responded partially to high glucose challenges from a db/db islet are shown in S3 Fig, and the movie of the same dysfunctional α and β cell is shown in S8 Movie. Overall, the diabetic mouse islet cells showed more heterogeneous marker expression profiles and function compared to WT mouse islets. This finding demonstrates that under disease conditions, other cell types, such as α cells, influx calcium in response to high glucose. Therefore, marker expression profiles are important to consider when measuring single cell calcium responses.

### Human islets show responses to high glucose challenges but in a poorly synchronized manner

**Fig 5:**
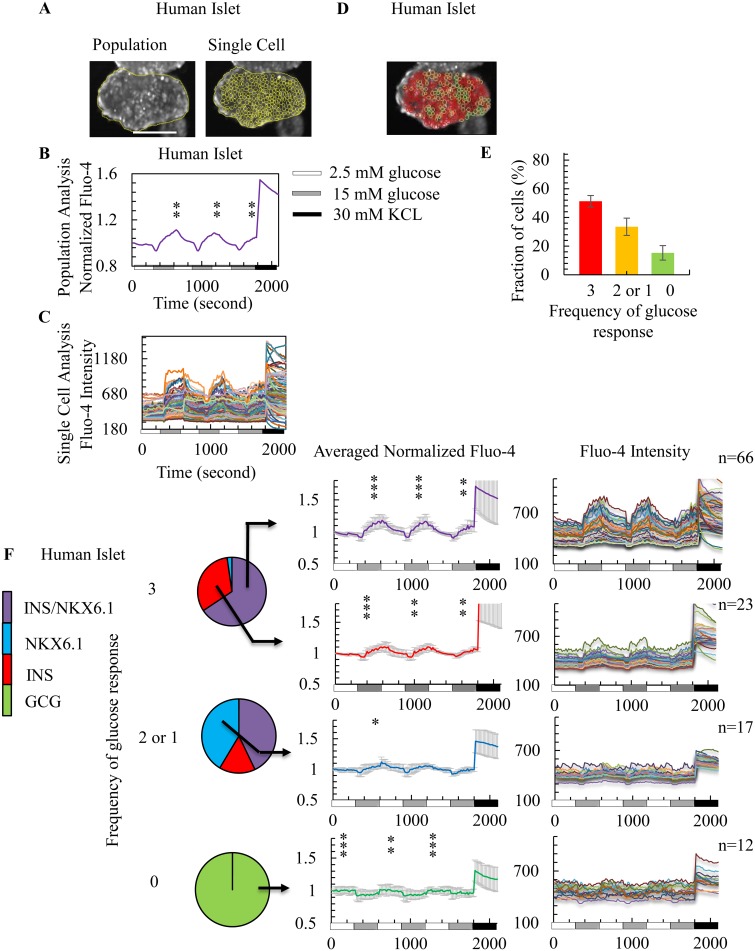
Human islets contain cells that influx calcium in response to multiple glucose challenges and show expression of β cell markers. (A) Representative images of analysis selection setting for population (left) and single cell (right) analysis for a human islet. Scale bar = 100 μm. (B) Representative population measurements of dynamic normalized Fluo-4 fluorescence intensity for one human islet (out of three islets analyzed). (C) Single cell measurements of dynamic Fluo-4 fluorescence intensity for human islets (from the same donor). (D) Representative images showing single cells that responded to 3 (red), 2 or 1 (orange), and 0 (green) glucose challenges in human islets. (E) Quantification of the frequency of cells responding to 15 mM glucose analyzed from 3 human islets (total number of cells analyzed from each islet was n = 245, n = 201, and n = 176). On average, WT human islets had 51±4% that responded 3 times, 33±6% that responded 2 or 1 times, and 15±5% that responded to no glucose challenge. (F) Marker expression profiles and responsiveness to glucose for individual cells of human islets. Color scheme is the same as Fig 4.

Calcium imaging during multiple glucose challenges was performed on intact human islets which were subsequently stained for marker expression (Fig 5). Population and single cell calcium imaging of human islets show responses to high glucose but with less synchronized responses compared to that of WT mouse islets (Fig 5A and 5B, 5C and S9 Movie). Similar to WT mouse islets, human islets that responded to all 3 high glucose stimulations were mostly β cells, as determined by INS/NKX6.1 coexpression. Cells that did not respond to high glucose showed an inversed calcium response to low glucose and expressed α cell markers (Fig 5D, 5E, and 5F). Human islets also contained dysfunctional β cells that showed partial calcium responses to glucose challenges with lower calcium influx peaks compared to fully calcium responsive cells (Fig 5F). Unlike WT mouse islets, human islets contained cells expressing INS marker alone and these showed three significant responses to high glucose challenges, albeit with lower calcium peaks than the response of double positive, INS/NKX6.1 human β cells.

## Discussion

The technique of calcium imaging sectioned islets during a high glucose challenge allowed visualization of the spread of a calcium wave among beta cells, demonstrating a connectivity of beta cells in the islet. [24, 25]. The same technique was used to show the calcium connectivity of beta cells residing within the first and second layers of intact islets [26]. These impressive studies demonstrated the coordinated β-to-β cell connections by analyzing the spread of a calcium wave from one end of the islet to the other during a single high glucose challenge. We have extended this approach by developing methods for evaluating islet function by measuring the response to multiple high glucose challenges for the whole islet population and for single cells. Islet function was analyzed by measuring the number of times a cell responds to stimuli that cause fluxes in intracellular calcium, thereby providing a quantitative assessment at the single cell level of the response of an islet cell to glucose. In addition, we developed methods to stain for cell markers on these same cells to compare gene expression with function (calcium imaging). Measuring calcium flux and determining marker expression in the same cells makes it possible to compare functional / dysfunctional cells with their gene expression. It is worth noting that although oscillations in insulin secretion have been reported when calcium influx is not oscillating ([32]), under most conditions the oscillations in calcium influx act as the principal driver of islet insulin oscillations.

In previous studies that used calcium imaging to test islet function, β cells were defined as the cells that showed calcium influx response to high glucose challenges, while α cells were defined as the cells with basal calcium binding but no response to high glucose. Applying this analytic approach to the studies reported here and focusing on the first glucose challenges only, a high number of β cells is observed (about 95%) and only a few α cells (about 5%) in WT mouse islets. Human islets would be scored as having a lower number of β cells (about 80%) and higher number of α cells (about 20%). These percentages are in alignment with previous reports using calcium imaging only [5, 24, 25]. However, if the response to all three high glucose challenges as well as marker expression profiles for each islet cell is considered, more information is uncovered about the cells. Using this comprehensive approach, WT mouse islet cells that influx calcium to all three high glucose challenges and express markers of β cells (INS/NKX6.1) consist of about 50% of calcium dye loaded cells which is consistent with studies showing these markers are essential for maintaining the functional and molecular traits of mature β cells [1]. WT mouse islet cells that did not respond to any high glucose challenges showed an inverse calcium trace, responding only to low glucose challenges. These cells expressed the α cell marker, GCG, and consisted of about 5% of measured cells.

It has been reported that calcium influx in α cells is necessary for GCG secretion at low glucose. However, the suppression of GCG secretion during high glucose stimulation is not mediated by shutting down of the calcium influx but rather through a calcium independent inhibitory pathway [9]. At least two significant decreases in calcium influx to high glucose challenges in WT mouse islet α cells were observed, as shown in S2 Fig and S7 Movie, perhaps due to the use of glucose stimulation range between 2.5mM-15mM rather than 1mM-12mM. Overall, no significant calcium responses to high glucose were observed in α cells, which is in line with other reports on α cell function [20–23, 33, 34]. Some β cells (INS/NKX6.1, ~10%) were observed in WT mouse islets that only responded to 1 or 2 of the 3 high glucose challenges. The method reported here, which examines the robustness of islet cell function using multiple glucose challenges, identifies varying functional phenotypes of INS/NKX6.1 co-expressing cells, even in WT mouse islets and could be an indicator of overall islet health or condition.

Continuing this theme, by determining islet cell identity of diabetic islets based solely on the calcium flux profile of the first glucose challenge, the db/db islets contain about 60% β cells and 40% α cells, while the NOD islets contain 75% β cells and 25% α cells. When characterizing the diabetic islets with the methods described here (multiple glucose challenges and marker staining), db/db and NOD mouse islets had no or very few functional β cells: 0% in db/db and about 5% in NOD. The diabetic mouse islets showed a high proportion of dysfunctional β cells that did not respond at all to high glucose stimulations. Importantly, the lack of third and sometime second and third responses to high glucose challenges in diabetic islets may be due to an overall lower amplitude of the calcium responses in diabetic islets in comparison to WT mouse islets. It is possible that minor calcium responses occur at the second and third challenge do not reach statistical significance. Regardless, the lack of detectable responses to these challenges highlights the dysfunction of these β cells.

The diabetic islets also contained dysfunctional α cells that expressed GCG and fluxed calcium in response to high glucose. One explanation for the appearance of these dysfunctional α cells could be inaccuracy of single cell selection. Single cells were selected by circling the cell based on the calcium signal and corresponding staining with the best intention of covering the cell without including neighboring cells. However, not all the cells are shaped as perfect circles. Furthermore, Fluo-4 dye only penetrates the first two cell layers of the islets making the cell selection more challenging. At the same time, inversed calcium signals from GCG positive cells were observed from WT and diabetic mouse islets indicating no or minimal signal contamination from neighboring β cells. Thus, in most cases single cell selections are targeted properly and the GCG positive α cells showing abnormal calcium response represent dysfunctional α cells rather than technical artifacts. It is possible that the dysfunctional α cells that demonstrate a calcium response more similar to that of β cells are transitioning into β cell-like cells. In support of this idea, Liang et al found that the α-cells from STZ induced diabetic rat islet α-cells dedifferentiated into precursor cells and has suggested these cells are candidates for β cell formation [35]. Further investigation is needed to confirm the state changes in these cells.

The reduced response of the human islets could be a result of the declining condition of the human islets, as they are typically received 5–10 days after the donor has deceased. Other factors, such as multiple sizes of cells or inherent variability in cells of human islets could affect the level of response observed [8, 36]. Nevertheless, similar marker and calcium influx pattern were observed in both human and WT mouse islets. One difference to note is that unlike what WT mouse islets, a fraction of INS positive cells (NKX6.1 negative) showed three responses to glucose challenges. The lack of NKX6.1 expression in this population of glucose responsive cells requires further investigation.

Overall, our method allows comparison of gene expression profiles to functional properties of individual cells of the islet. This method allows characterization of WT and disease mouse islet function and can be used for the study of human islets. As such, this method will be valuable for qualifying β cells obtained from various sources, such as stem cell-derived β cells, as functional, bona fide β cells and for understanding β cell dysfunction in diabetes.

## Supporting Information

S1 Fig INS, GCG, and NKX6.1 immunofluorescence staining images of WT, db/db, NOD mouse islets, and human islets. (A-D) Representative immunofluorescence images of (A) WT mouse islets, (B) db/db mouse islets, (C) NOD mouse islets, and (D) human islets labeled for INS (left), GCG (middle), and NKX6.1 (right).
(TIF)

S2 Fig Fully calcium responsive cells co-express NKX6.1/INS while inversely responsive cells express GCG in WT mouse islets. Top panel of images: Two cells were labeled with arrows and circles either in green (no calcium response to three high glucose challenges) or in red (calcium responses to all three challenges). Green labeled cell was an INS negative, GCG positive, and NKX6.1 negative cell which we can identify as α cell. The red labeled cell was an INS positive, GCG negative, and NKX6.1 positive cell which we can identify as a β cell. The representative merged images, INS/NKX6.1/GCG, NKX6.1/GCG, and INS/GCG of these labeled cells are shown in S4A Fig. Bottom panel of graphs: (A) Graph representing the measurements of dynamic normalized Fluo-4 fluorescence intensity for the healthy α cell indicated by GCG positive immuno-fluorescence staining. (B) Graph representing the measurements of dynamic normalized Fluo-4 fluorescence intensity for the healthy β cell indicated by INS/NKX6.1 co-positive immuno-fluorescence staining.
(TIF)

S3 Fig db/db mouse islets contain dysfunctional α and β cells. Top panel of images: Two cells were labeled with arrows and circles in orange to indicate that both cells partially responded to three high glucose challenges. The left cell (labeled A) was a GCG positive, INS/NKX6.1 negative α cell. The right cell (labeled B) was β cell with INS/NKX6.1 co-expression. The representative merged images, INS/NKX6.1/GCG, NKX6.1/GCG, and INS/GCG of these labeled cells were shown in S4B Fig. Bottom panel of graphs: (A) Graph representing the measurements of dynamic normalized Fluo-4 fluorescence intensity for the dysfunctional α cell indicated by GCG positive immuno-fluorescence staining. (B) Graph representing the measurements of dynamic normalized Fluo-4 fluorescence intensity for the dysfunctional β cell indicated by INS/NKX6.1 co-positive immuno-fluorescence staining.
(TIF)

S4 Fig Immunofluorescence staining images of intact WT and db/db mouse islet cells. (A) Immunofluorescence staining of intact WT mouse islet cell. Top panel shows merged, immunostained images (INS/GCG/NKX6.1, NKX6.1/GCG, and INS/GCG from left to right) of a healthy α cell. Bottom panel shows merged, immunostained images (INS/GCG/NKX6.1, NKX6.1/GCG, and INS/GCG from left to right) of a healthy β cell. (B) Immunofluorescence staining of intact db/db mouse islet cell. Top panel shows merged, immunostained images (INS/GCG/NKX6.1, NKX6.1/GCG, and INS/GCG from left to right) of a dysfunctional α cell. Bottom panel shows merged, immunostained images (INS/GCG/NKX6.1, NKX6.1/GCG, and INS/GCG from left to right) of a dysfunctional β cell.
(TIF)

S1 Movie Movie of Fig 1C. Imaging of Fluo-4 calcium influx in the intact WT mouse islets. Islets were imaged while being stimulated with a series of sequential glucose challenges: low (2.5 mM)-high (15 mM)-low-high-low-high followed by KCl depolarization. Islets were imaged at 30X objective for population analysis. Eighteen images were taken during each 5 minutes of glucose challenge, and 126 images total were acquired for each islet. Images were made in to a stack and converted to a movie (10 frames per second).
(ZIP)

S2 Movie Movie of Fig 1D. Imaging of Fluo-4 calcium influx in the intact WT mouse islets. Islets were challenges with 5 minutes of 2.5 mM, 25 minutes of 15 mM, and 5 minutes of 30 mM KCl. Imaging was done as in S1 Movie.
(ZIP)

S3 Movie Movie of Fig 2A. Imaging of Fluo-4 calcium influx in the intact WT mouse islet. Islets were imaged while being stimulated with a series of sequential glucose challenges: low (2.5 mM)-high (15 mM)-low-high-low-high followed by KCl depolarization. Islets were imaged at 70X objective for single cell analysis. 18 images were taken during each 5 minutes of glucose challenge and 126 images total were acquired for each islet. Images were made in to a stack and converted to a movie (10 frames per second).
(ZIP)

S4 Movie Movie of Fig 2B. Imaging of Fluo-4 calcium influx in the intact T2D db/db mouse islet. Imaging was done as in S3 Movie.
(7Z) Click here for additional data file.

S5 Movie Movie of Fig 2C. Imaging of Fluo-4 calcium influx in the intact T1D NOD mouse islet. Imaging was done as in S3 Movie.
(ZIP)

S6 Movie Movie of S2A Fig. Imaging of Fluo-4 calcium influx in the intact WT mouse islet healthy α cell indicated by GCG positive immuno-fluorescence staining. Imaging was done as in S3 Movie.
(AVI)

S7 Movie Movie of S2B Fig. Imaging of Fluo-4 calcium influx in the intact WT mouse islet healthy β cell indicated by INS/NKX6.1 co-positive immuno-fluorescence staining. Imaging was done as in S3 Movie.
(AVI)

S8 Movie Movie of S3 Fig. Imaging of Fluo-4 calcium influx in the intact T2D db/db mouse islet dysfunctional α cell (A) and dysfunctional β cell (B) indicated by GCG positive or INS/NKX6.1 co-positive immuno-fluorescence staining respectively. Imaging was done as in S3 Movie.
(AVI)

S9 Movie Movie of Fig 4A. Imaging of Fluo-4 calcium influx in the intact human islet. Imaging was done as in S3 Movie.
(AVI)

S1 Table Quantification of the number of different glucose responsive cells and their marker expression in mouse and human islets. Frequency of glucose responses and the corresponding marker expression in WT mouse islets, db/db mouse islets, NOD mouse islets, and WT human islets were listed as follows: the total cell number that showed each marker and % of cell number of each marker within the indicated glucose responsiveness over the total cell number of the indicated glucose responsiveness. Three islets per genotype were analyzed for marker expression.
(TIF)

## References

[1] Taylor BL, Liu FF, Sander M. Nkx6.1 Is Essential for Maintaining the Functional State of Pancreatic Beta Cells. Cell Report. 2013 v4, 5, 1262–1275. 10.1016/j.celrep.2013.08.010 PMC4058003 24035389

[2] Hrvatin S, Deng F, O’Donnell CW, Gifford DK, Melton DA. MARIS: Method for Analyzing RNA following Intracellular Sorting. PLOSone. 2014;10 1371. 10.1371/journal.pone.0089459 PMC3940959 24594682

[3] Carter JD, Dula SB, Corbin KL, Wu R, Nunemaker CS. A practical guide to rodent islet isolation and assessment. Biol Proced Online. 2009 11:3–31. 10.1007/s12575-009-9021-0 19957062 PMC3056052

[4] Blum B, Hrvatin S, Schuetz C, Bonal C, Rezania A & Melton DA. Functional beta-cell maturation is marked by an increased glucose threshold and by expression of urocortin 3. Nature Biotechnology. 2012 30, 2610264 10.1038/nbt.2141 PMC4617627 22371083

[5] Marchetti P, Scharp DW, McLear M, Gingerich R, Finke E, Olack B, et al Pulsatile insulin secretion from isolated human pancreatic islets. Diabetes. 1994 6; 43 (6):827–30. 8194670 10.2337/diab.43.6.827

[6] Sweet IR, Gilbert M. Contribution of Calcium Influx in Mediating Glucose-Stimulated Oxygen Consumption in Pancreatic Islets. Diabetes. 2006 55, 3509–3519. 17130499 10.2337/db06-0400

[7] Merrins MJ, Fendler B, Zhang M, Sherman A, Bertram R, Satin LS. Metabolic Oscillations in Pancreatic Islets Depend on the Intracellular Ca2+ Level but Not Ca2+ Oscillations. Biophysical Journal. 2010 99, 76–84. 10.1016/j.bpj.2010.04.012 20655835 PMC2895383

[8] Mohammed JS, Wang Y, Harvat TA, Oberholzer J, Eddington DT. Microfluidic device for multimodal characterization of pancreatic islets. The Royal Society of Chemistry. 2008 9, 97–106. 10.1039/b809590f PMC3759253 19209341

[9] Hamming KS, Soliman D, Webster NJ, Searle GJ, Matemisz LC, Liknes DA, et al Inhibition of beta cell Sodium-Calcium Exchange Enhances Glucose-Dependent Elevations in Cytoplasmic Calcium and Insulin Secretion. Diabetes. 2010 59, 1686–1693. 10.2337/db09-0630 20413506 PMC2889768

[10] Nunemaker CS, Zhang M, Wasserman DH, McGuinness OP, Powers AC, Bertram R, et al Individual mice can be distinguished by the period of their islet calcium oscillations: is there an intrinsic islet period that is imprinted in vivo? Diabetes. 2005 54: 3517–3522. 16306370 10.2337/diabetes.54.12.3517

[11] Speier S, Rupnik M. A novel approach to in situ characterization of pancreatic ß-cells. Pflügers Archiv European Journal of Physiology. 2003 446: 553–558. 10.1007/s00424-003-1097-9 12774232

[12] Rupnik M. The physiology of rodent beta-cells in pancreas slices. Acta Physiologica. 2009 195: 123–138. 10.1111/j.1748-1716.2008.01927 18983446

[13] Sánchez-Cárdenas C, Hernández-Cruz A. GnRH-Induced Ca^2+^-Signalling Patterns in Mouse Gonadotrophs Recorded from Acute Pituitary Slices in vitro. Neuroendocrinology. 2010 91: 239–255. 10.1159/000274493 20090289

[14] Hodson DJ, Schaeffer M, Romano N, Fontanaud P, Lafont C, Birkenstock J, et al Existence of long-lasting experience-dependent plasticity in endocrine cell networks. Nat Communications. 2012 3: 605 10.1038/ncomms1612 22215080 PMC3272579

[15] Schneggenburger R, Forsythe ID. The calyx of Held. Cell and Tissue Research. 2006 326: 311–337. 10.1007/s00441-006-0272-7 16896951

[16] Moser T, Neher E. Rapid Exocytosis in Single Chromaffin Cells Recorded from Mouse Adrenal Slices. The Journal of Neuroscience. 1997 17: 2314–2323. 9065492 10.1523/JNEUROSCI.17-07-02314.1997 PMC6573505

[17] Edwards FA, Konnerth A, Sakmann B, Takahashi T. A thin slice preparation for patch clamp recordings from neurons of the mammalian central nervous system. Pflügers Archiv European Journal of Physiology. 1989 414: 600–612. 10.1007/bf00580998 2780225

[18] García AG, García-De-Diego AM, Gandía L, Borges R, García-Sancho J. Calcium Signaling and Exocytosis in Adrenal Chromaffin Cells. Physiological Reviews. 2006 86: 1093–1131. 10.1152/physrev.00039.2005 17015485

[19] Sedej S, Rose T, Rupnik M. cAMP increases Ca ^2+^ -dependent exocytosis through both PKA and Epac2 in mouse melanotrophs from pituitary tissue slices. J Physiol. 2005 567: 799–813. 10.1113/jphysiol.2005.090381 PMC1474225 15994184

[20] Asada N. Shibuya I, Iwanaga T, Niwa K, Kanno T. Identification of alpha- and beta-cells in intact isolated islets of Langerhans by their characteristic cytoplasmic Ca2+ concentration dynamics and immunocytochemical staining. Diabetes. 1998 47: 751–757. 9588446 10.2337/diabetes.47.5.751

[21] Nadal A, Quesada I, Soria B. Homologous and heterologous asynchronicity between identified alpha-, beta-, and delta-cells within intact islets of Langerhans in the mouse. J Physiol. 1999 517: 85–93. 10226151 10.1111/j.1469-7793.1999.0085z.x PMC2269319

[22] Quesada I, Nadal A, Soria B. Different effects of tolbutamide and diazoxide in alpha, beta-, and dela-cells within intact islets of Langerhands. Diabetes. 1999 48: 2390–2397. 10580428 10.2337/diabetes.48.12.2390

[23] Quesada I, Todorova MG, Alonso-Magdalena P, Beltra M, Carneiro EM, Martin F, et al Glucose Induced Opposite Intracellular Ca2+ Concentration Oscillatory Patterns in Identified alpha- and beta-cells Within Intact Human Islets of Langerhans, Diabetes. 2006 55: 2463–2469. 16936194 10.2337/db06-0272

[24] Stozer A, Dolensek J, Rupnik MS. Glucose-Stimulated Calcium Dynamics in Islets of Langerhans in Acute Mouse Pancreas Tissue Slices. PLoS ONE. 2013 v8, 1, e54638, 1–13. 10.1371/journal.pone.0054638 23358454 PMC3554663

[25] Stozer A, Gosak M, Dolensek J, Perc M, Marhl M, Rupnik MS, et al Functional Connectivity in Islets of Langerhands from mouse Pancreas Tissue Slices. PLoS ONE. 2013 v9, 2, e1002923, 1–12. 10.1371/journal.pcbi.1002923 23468610 PMC3585390

[26] Hodson DJ, Mitchell RK, Bellomo EA, Sun G, Vinet L, Meda P, et al Lipotoxicity disrupts incretin-regulated human beta cell connectivity. J Clin Invest. 2013 123 (10): 4182–4194. 10.1172/JCI68459 24018562 PMC4382273

[27] Li DS, Yuan YH, Tu HJ, Liang QL, Dai LJ. A protocol for islet isolation from mouse pancreas. Nat. Protoc. 2009 4:1649–1652. 10.1038/nprot.2009.150 19876025

[28] Gilon P, Shepherd RM, Henquin JC. Oscillations of secretion driven by oscillations of cytoplasmic Ca2+ as evidences in single pancreatic islets. J. Biol. Chem. 1993 268:22265–22268. 8226733

[29] Henquin JC. Ionic and metabolic messengers in the control of insulin secretion. Jpn. J. Physiol. 1997 47(Suppl. 1):S9 9266308

[30] Kjems LL, Ravier MA, Jonas JC, Henquin JC. Do oscillations of insulin secretion occur in the absence of cytoplasmic Ca2+ oscillations in beta-cells? Diabetes. 2002 51(Suppl. 1):S177–S182. 11815478 10.2337/diabetes.51.2007.s177

[31] Wang RN, Rosenberg L. Maintenance of beta-cell function and survival following islet isolation requires re-establishment of the islet-matrix relationship. J of Endocrinology. 1999 163: 181–190. 10556766 10.1677/joe.0.1630181

[32] Westerlund J, Gylfe E, Bergsten P. Pulsatile insulin release from pancreatic islets with nonoscillatory elevation of cyto-plasmic Ca2+. J. Clin. Invest. 1997 100:2547–2551. 9366569 10.1172/JCI119797 PMC508455

[33] Sylvain J, Marchand L, Piston DW. Glucose decouples intracellular Ca2+ activity from glucagon secretion in mouse pancreatic islet alpha-cells. PLoS ONE. 2012 v7, 10, e47084, 1–10. 10.1371/journal.pone.0047084 23077547 PMC3471958

[34] Le Marchand SJ, Piston DW. Glucose suppression of glucagon secretion metabolic and calcium responses from alpha-cells in intact mouse pancreatic islets. JBC. 2010 1–20. 10.1074/jbc.M109.069195 PMC2863245 20231269

[35] Liang XD, Guo YY, Sun M, Ding Y, Wang N, Yuan L, et al Streptozotocin-induced expression of Ngn3 and Pax4 in neonatal rat pancreatic α-cells. World Journal of Gastroenterology. 2011 vol. 17, no. 23, pp. 2812–2820. 10.3748/wjg.v17.i23.2812 21734788 PMC3120940

[36] Rutter GA, Hodson DJ. Minireview: intra islet regulation of insulin secretion in humans. Mol Endocrinol. 2013 12: 1984–95. 10.1210/me.2013-1278 PMC5426601 24243488

