# Supplementary figures and images for: Testing Pancreatic Islet Function at the Single Cell Level by Calcium Influx with Associated Marker Expression
Source: PLoS One. 2015 Apr 8;10(4):e0122044. doi: 10.1371/journal.pone.0122044 (PMC4390334; doi:10.1371/journal.pone.0122044)

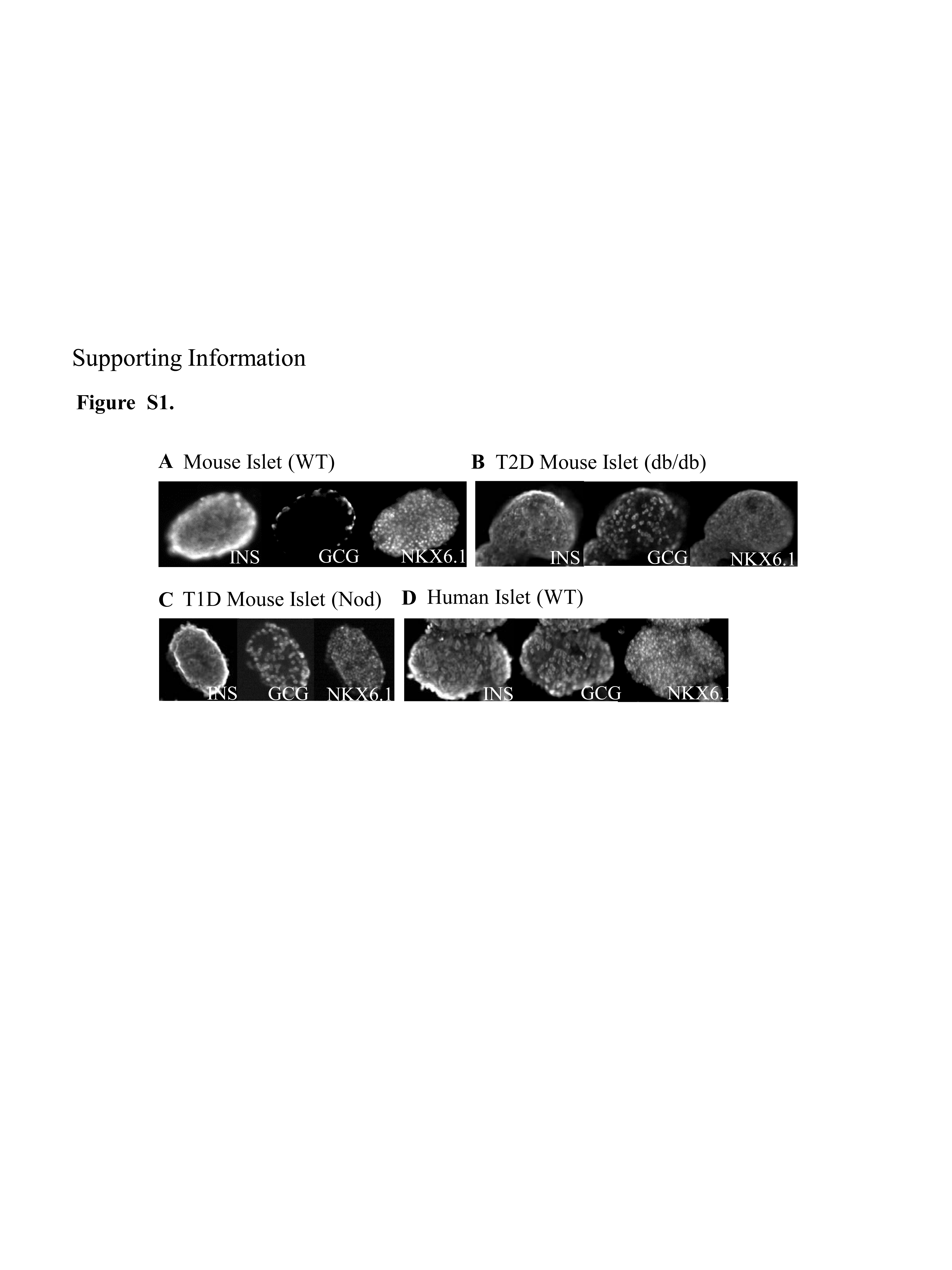

Supplement: S1 Fig — (A-D) Representative immunofluorescence images of (A) WT mouse islets, (B) db/db mouse islets, (C) NOD mouse islets, and (D) human islets labeled for INS (left), GCG (middle), and NKX6.1 (right). (TIF) [file pone.0122044.s001.tif]

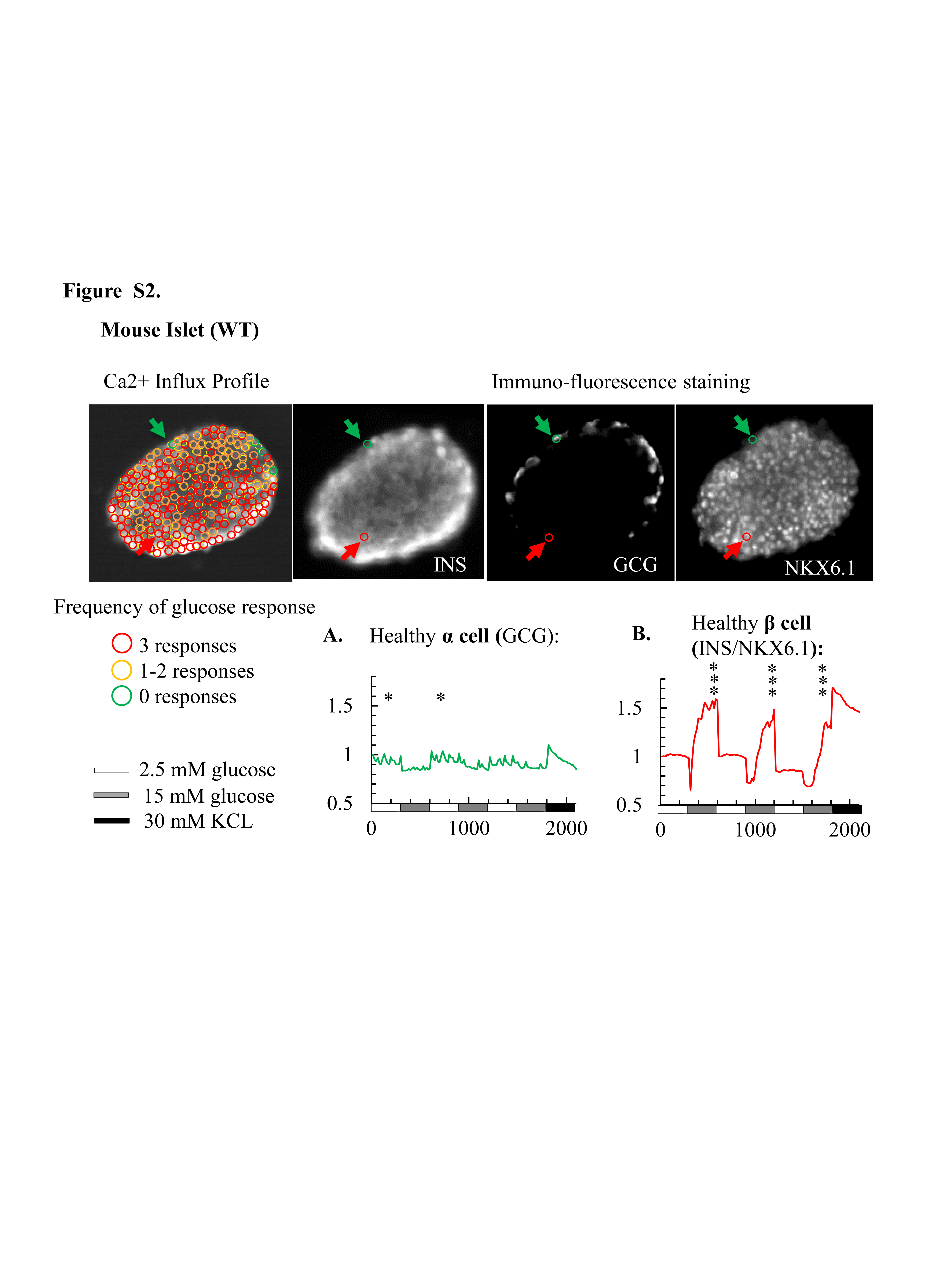

Supplement: S2 Fig — Top panel of images: Two cells were labeled with arrows and circles either in green (no calcium response to three high glucose challenges) or in red (calcium responses to all three challenges). Green labeled cell was an INS negative, GCG positive, and NKX6.1 negative cell which we can identify as α cell. The red labeled cell was an INS positive, GCG negative, and NKX6.1 positive cell which we can identify as a β cell. The representative merged images, INS/NKX6.1/GCG, NKX6.1/GCG, and INS/GCG of these labeled cells are shown in S4A Fig. Bottom panel of graphs: (A) Graph representing the measurements of dynamic normalized Fluo-4 fluorescence intensity for the healthy α cell indicated by GCG positive immuno-fluorescence staining. (B) Graph representing the measurements of dynamic normalized Fluo-4 fluorescence intensity for the healthy β cell indicated by INS/NKX6.1 co-positive immuno-fluorescence staining. (TIF) [file pone.0122044.s002.tif]

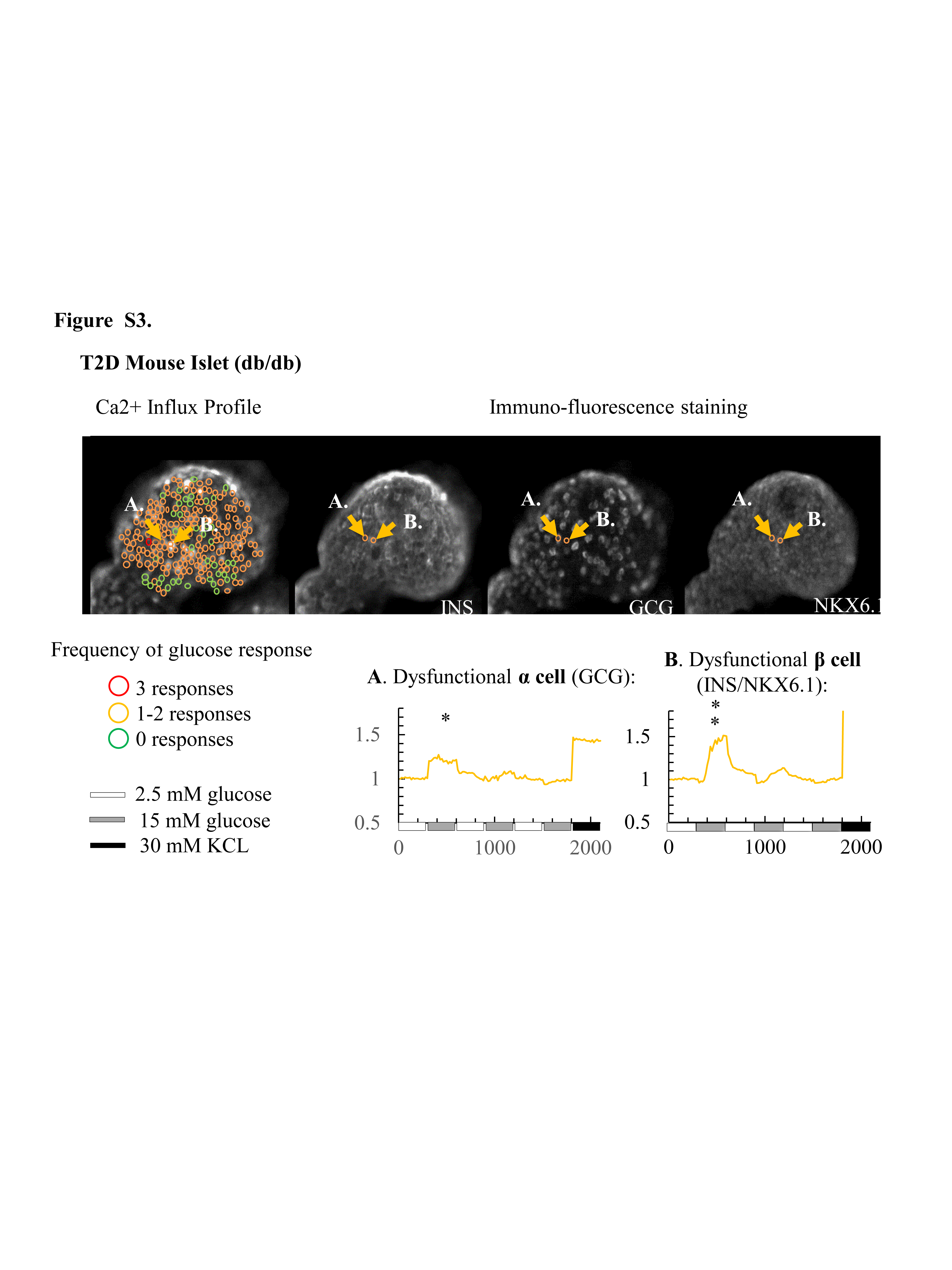

Supplement: S3 Fig — Top panel of images: Two cells were labeled with arrows and circles in orange to indicate that both cells partially responded to three high glucose challenges. The left cell (labeled A) was a GCG positive, INS/NKX6.1 negative α cell. The right cell (labeled B) was β cell with INS/NKX6.1 co-expression. The representative merged images, INS/NKX6.1/GCG, NKX6.1/GCG, and INS/GCG of these labeled cells were shown in S4B Fig. Bottom panel of graphs: (A) Graph representing the measurements of dynamic normalized Fluo-4 fluorescence intensity for the dysfunctional α cell indicated by GCG positive immuno-fluorescence staining. (B) Graph representing the measurements of dynamic normalized Fluo-4 fluorescence intensity for the dysfunctional β cell indicated by INS/NKX6.1 co-positive immuno-fluorescence staining. (TIF) [file pone.0122044.s003.tif]

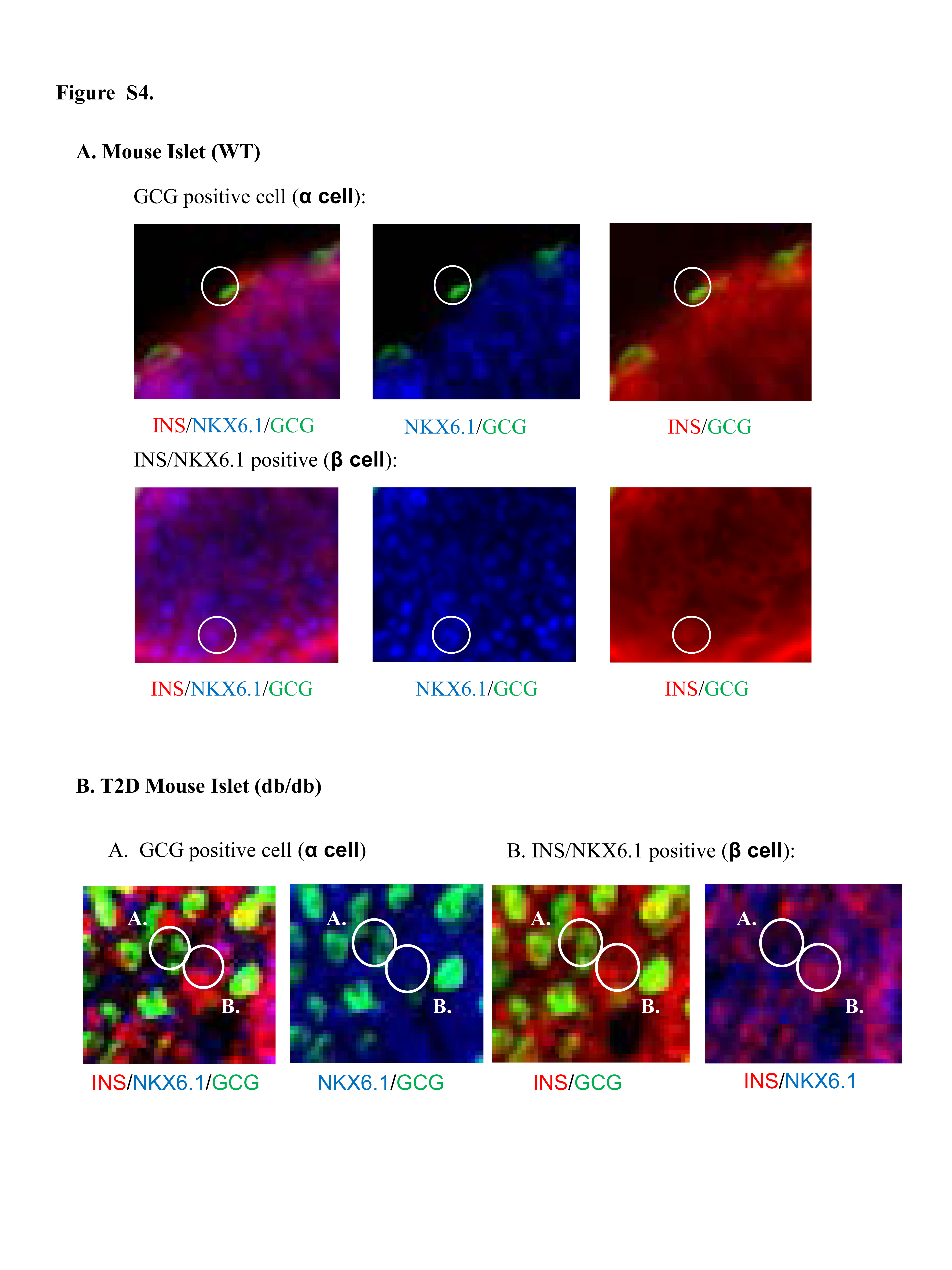

Supplement: S4 Fig — (A) Immunofluorescence staining of intact WT mouse islet cell. Top panel shows merged, immunostained images (INS/GCG/NKX6.1, NKX6.1/GCG, and INS/GCG from left to right) of a healthy α cell. Bottom panel shows merged, immunostained images (INS/GCG/NKX6.1, NKX6.1/GCG, and INS/GCG from left to right) of a healthy β cell. (B) Immunofluorescence staining of intact db/db mouse islet cell. Top panel shows merged, immunostained images (INS/GCG/NKX6.1, NKX6.1/GCG, and INS/GCG from left to right) of a dysfunctional α cell. Bottom panel shows merged, immunostained images (INS/GCG/NKX6.1, NKX6.1/GCG, and INS/GCG from left to right) of a dysfunctional β cell. (TIF) [file pone.0122044.s004.tif]

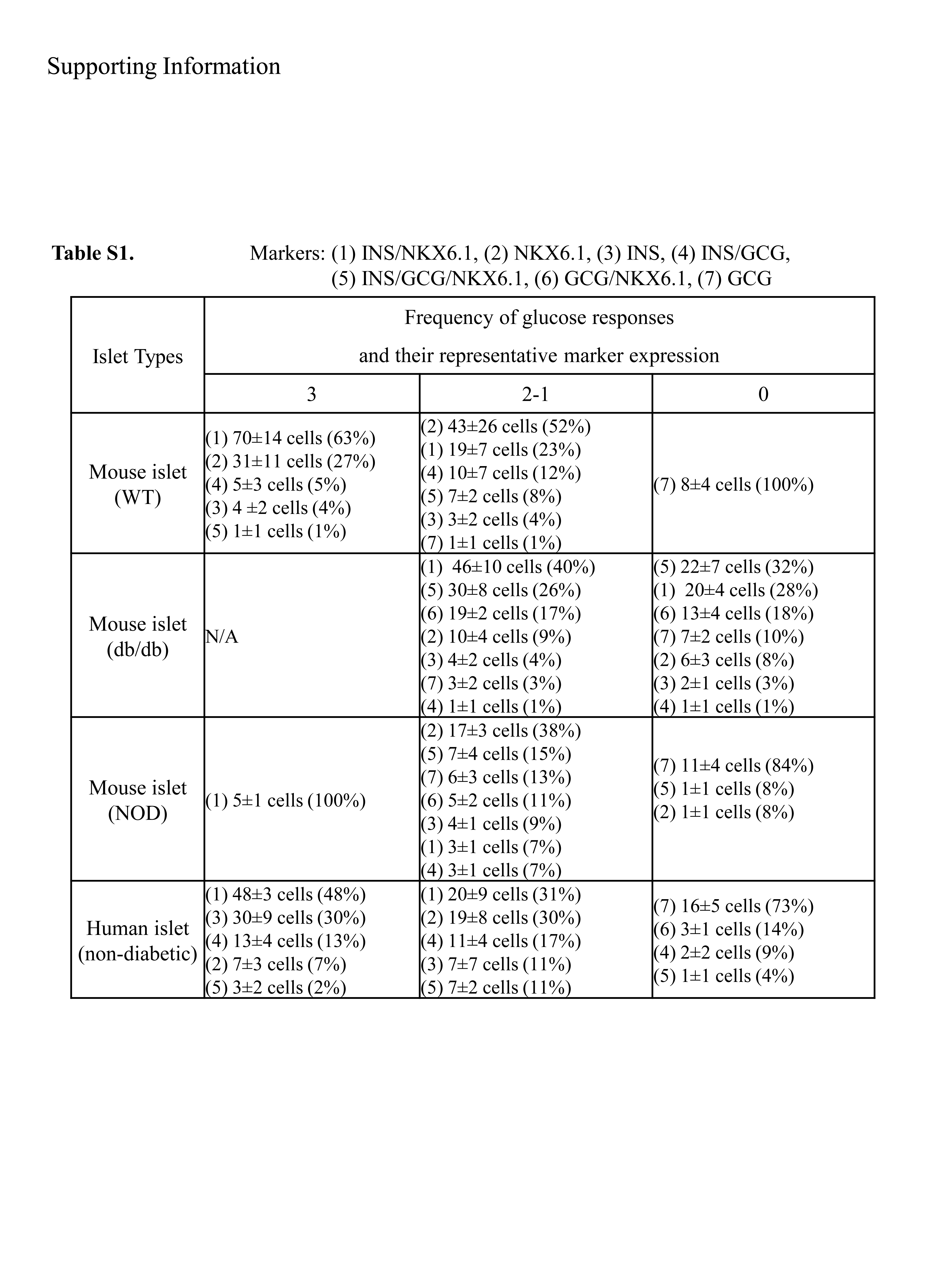

Supplement: S1 Table — Frequency of glucose responses and the corresponding marker expression in WT mouse islets, db/db mouse islets, NOD mouse islets, and WT human islets were listed as follows: the total cell number that showed each marker and % of cell number of each marker within the indicated glucose responsiveness over the total cell number of the indicated glucose responsiveness. Three islets per genotype were analyzed for marker expression. (TIF) [file pone.0122044.s014.tif]
